# Associations Between Waiting Times, Service Times, and Patient Satisfaction in an Endocrinology Outpatient Department: A Time Study and Questionnaire Survey

**DOI:** 10.1177/0046958017739527

**Published:** 2017-11-22

**Authors:** Zhenzhen Xie, Calvin Or

**Affiliations:** 1 The University of Hong Kong, China

**Keywords:** patient wait times, patient satisfaction, time and motion study, cross-sectional study, survey and questionnaire, public health systems research

## Abstract

The issue of long patient waits has attracted increasing public attention due to the negative effects of waiting on patients’ satisfaction with health care. The present study examined the associations between actual waiting time, perceived acceptability of waiting time, actual service time, perceived acceptability of service time, actual visit duration, and the level of patient satisfaction with care. We conducted a cross-sectional time study and questionnaire survey of endocrinology outpatients visiting a major teaching hospital in China. Our results show that actual waiting time was negatively associated with patient satisfaction regarding several aspects of the care they received. Also, patients who were less satisfied with the sociocultural atmosphere and the identity-oriented approach to their care tended to perceive the amounts of time they spent waiting and receiving care as less acceptable. It is not always possible to prevent dissatisfaction with waiting, or to actually reduce waiting times by increasing resources such as increased staffing. However, several improvements in care services can be considered. Our suggestions include providing clearer, more transparent information to keep patients informed about the health care services that they may receive, and the health care professionals who are responsible for those services. We also suggest that care providers are encouraged to continue to show empathy and respect for patients, that patients are provided with private areas where they can talk with health professionals and no one can overhear, and that hospital staff treat the family members or friends who accompany patients in a courteous and friendly way.

## Introduction

Long waiting times for patients are commonly seen in outpatient facilities, and this difficulty contributes to a range of public health issues, including impaired access to care, interruption of hospital work patterns, and patient dissatisfaction.^1,2^ The Canadian Institute for Health Information reported that for 90% of the visits to Canadian emergency departments, the actual time spent from triage to the doctor’s initial assessment was much longer than the recommended response time. The actual waiting times were 47 to 229 minutes, as compared with the recommended standard of up to 120 minutes.^3^ In China, a study among outpatients in a tertiary hospital showed that the average wait time for registration was 98 minutes and that some patients waited as many as 13.5 hours to ensure a registration with a certain doctor.^4^ It is common to find patient care being postponed because of long waiting periods, and patients often spend more time waiting than actually consulting with health care providers. For instance, a national study of Malaysian public hospitals documented that the average patient wait time, from registration to receipt of a prescription slip, was more than 2 hours, whereas the average time spent consulting the medical personnel was just 15 minutes.^5^ A study in a US tertiary hospital showed that 61% of the patients waited 90 to 180 minutes in the outpatient department, while 36.1% spent less than 5 minutes with the doctor in the consulting room.^6^

## Background

Among the several public health issues that might be related to long patient waits, the problem of patient dissatisfaction with care has attracted the most attention. Studies in various settings, such as military outpatient clinics,^7^ outpatient veterans administration clinics,^8^ outpatient primary care units,^9^ ambulatory services,^10^ outpatient ophthalmology clinics,^11^ outpatient orthopedic clinics,^12^ or university health service clinics^13-16^ have all demonstrated that patient waits are associated with various levels of patient dissatisfaction. For example, Bar-dayan et al^7^ found that clinic waiting time was a major determinant of dissatisfaction among soldiers seeking medical services. Probst et al^16^ showed that patients were more likely to be satisfied if they did not have to wait long. Camacho et al^9^ found that increased waits resulted in reduced patient satisfaction and decreased willingness to return. Dansky and Miles^13^ found that patient satisfaction decreased when the time waiting to see a clinician increased. Huang et al^17^ focusing on a Chinese population found that emergency department patients who were less satisfied with the amount of time that they spent waiting were less likely to be satisfied with the overall care services that they received.

Patient waits were also found to be associated with the patients’ perceptions regarding other aspects of care that were not directly related to satisfaction with medical care. For instance, Spaite et al^18^ discovered that patients were more likely to perceive the staff as kind or compassionate if they waited for a shorter time. Bleustein et al^19^ found that longer waits could diminish the patients’ perception of the doctors’ capability and decrease the patients’ confidence in the health services provided. De Man et al^20^ found that patient wait times had a great effect on the patients’ perceptions regarding the ability of their caregivers to perform health services reliably and accurately.

Considerable efforts have been made to understand the factors that cause long waits and to determine how this problem can be mitigated. The commonly identified causes include inadequate staffing, limited resources, high demand due to seasonal illnesses, and unnecessary visits to medical facilities. Strategies to reduce wait time and improve satisfaction with care have included the revamping of scheduling systems and better workforce management.^21,22^ However, due to shortages of staff and increases in patient volume, prolonged waits are often inevitable. Clearly, solutions to this demand-supply problem are difficult to solve with limited resources.

Some researchers have approached the issues of prolonged waiting and patient dissatisfaction from a psychological perspective. They have focused on mitigating patient dissatisfaction with long waits through methods related to perception and psychology. Such methods include managing patients’ expectations by advising them in advance about the time they should anticipate spending in the clinic,^12^ informing patients in the waiting room how much longer their wait is expected to be,^13^ keeping the patients occupied while they are waiting,^13^ providing clear instructions through public information systems,^15,23^ and providing the patients with health care education.^8^ Other studies have showed that the time spent with doctors is a more influential factor in patient satisfaction than the waiting time.^24^ For instance, one study showed that a longer time spent with the doctors could moderate the patients’ dissatisfaction with long waiting times.^14^ These researchers concluded that shortening patient waiting times at the expense of time spent with the patient would be counterproductive in terms of patient satisfaction.

As the problem of long waiting periods remains a common difficulty that can compromise patient satisfaction, it seems important to gain a better understanding of how the factors of waiting times, service times, and patient satisfaction with care are related. In this study, we examined this problem in an endocrinology outpatient department of a major teaching hospital in China. The problem of long waiting periods had not yet been thoroughly examined in this situation. The objectives of the study were to determine the associations between a number of time-related variables representing the patients’ waiting periods (ie, actual waiting time and perceived acceptability of waiting time), the care services (ie, actual service time and perceived acceptability of service time), and the hospital visits (ie, actual visit duration), with a view to examining how these variables influenced patient satisfaction. Through this study, we assessed the patient wait time issue in an endocrinology outpatient department, and explored alternatives for managing the patients’ perceptions of waiting time, service time, and satisfaction with care.

## Methods

The protocol of this study was approved by the Institutional Review Board of the University of Hong Kong/Hospital Authority Hong Kong West Cluster (reference number: UW13-145). We obtained the written informed consent of the participants before collecting any data.

### Sample and Procedure

The participants were recruited from an endocrinology outpatient department of a major (1700-bed) teaching hospital in China. Patients were eligible to participate in the study if they visited the department for health care during the study period, aged 18 years or older, and were able to understand the study protocol and respond to the study questions.

Two trained researchers (Da Tao and Jing Xu) were positioned in the lobby of the hospital and randomly approached patients who were visiting the outpatient department to solicit their participation. The researchers then introduced the study and determined the patients’ eligibility. They informed the patients that their participation was voluntary and that they could choose to withdraw from the study at any time without giving reasons. The eligible patients who agreed to participate then allowed the researchers to collect data by shadowing and observing them until they finished their visits to the department. To increase the reliability of the study, the researchers adopted an investigator triangulation method.^25^ In this method, each of the 2 researchers used a stopwatch and an information sheet to independently observe and record the start and end times for each care activity or waiting period that the patients experienced. These data were then cross-checked and verified prior to data analysis.

Using a questionnaire, the researchers collected the patients’ demographic information while they were waiting in the reception room (including their age, gender, self-reported health status, highest education level attained, and frequency of hospital visits). Then, at the end of the patients’ hospital visits, the researchers assessed the patients’ perceptions regarding their satisfaction with the quality of health care they received.

### Study Design

Our data collection was based on 2 methods. First, we performed an observational time study to measure 3 actual time outcomes: the patients’ actual waiting times, the actual service times, and the actual visit duration. Second, we conducted a questionnaire survey to evaluate the patients’ perceived acceptability of the waiting time and service time, and their satisfaction with the care received. Associations among the actual time outcomes, the perceived acceptability of time outcomes, and the levels of satisfaction were examined.

#### Time study and actual time outcomes

The 3 actual time outcomes were assessed through a time study. The definitions and measures used were identified according to the list of health care activities, as obtained from the standard outpatient care procedures provided by the hospital. These definitions and measures are presented below.

- *Actual waiting time* refers to the total number of minutes that each patient spent waiting during the hospital visit. This number was calculated as the sum of the time that the patient spent waiting for every health care service, as measured from the time when the patient registered to the time when he or she received the care service, or from the time when the patient finished one care service to the time when he or she started with the next service.
- *Actual service time* refers to the total number of minutes that a patient spent receiving health care services during the hospital visit. This number was calculated as the sum of the time that the patient spent receiving every health care service, with each service measured from the time when the patient started that service until the time when that service concluded.
- *Actual visit duration* refers to the total number of minutes that a patient spent in the hospital, which was measured from the time when the patient registered on arrival to the time when the patient completed care and left the hospital.

#### Questionnaire survey, perceived acceptability of time outcomes, and satisfaction with care

The questionnaire used to assess the patients’ satisfaction with the care had 3 scales, which have been found valid and internally reliable in previous studies.^26-29^ These scales were (1) the short form of the Quality From the Patient’s Perspective (QPP) questionnaire,^26^ (2) the Patient Satisfaction Questionnaire short-form (PSQ),^27^ and (3) the Visit-Specific Satisfaction Questionnaire (VSQ).^29,30^ These scales are presented in Tables A1, A2, and A3 in the “Appendix” section. The QPP questionnaire consisted of 22 items, which measured 18 aspects of care quality, in 4 dimensions. The items were rated on a 5-point scale, ranging from 1 (strongly disagree) to 5 (strongly agree). The score for each aspect was determined by the average score of the items that measured the aspect. The PSQ has 18 items, assessing 7 aspects of patient satisfaction. The items are rated on a 5-point scale, ranging from 1 (strongly disagree) to 5 (strongly agree). In a similar way, the score for each aspect was determined by the average score of the items that measured the aspect. The VSQ assesses 9 aspects of patient satisfaction, with each item measuring 1 aspect. The items are rated on a 5-point scale, ranging from 1 (poor) to 5 (excellent). We adopted the *QPP-treatment waiting time* and the *VSQ-waiting time* scales to indicate the patients’ perceived acceptability of waiting time, and the *PSQ-time spent with doctor* and *VSQ-service time* scales to indicate the patients’ perceived acceptability of service time.

### Data Analysis

Time data were consolidated to obtain the actual waiting times, actual service times, and actual visit durations for each participant. The patients’ responses for questionnaire items PSQ 4, 7, 9, 10, 12 to 14, 16, and 17 were reversed during the analysis, so that higher scores would represent higher patient satisfaction for those aspects. We computed the descriptive statistics (mean, standard deviation [SD], median, minimum [min], and maximum [max]) for all of the actual time outcomes and questionnaire items, to assess the waiting time problem and the patients’ satisfaction level. In addition, a correlation analysis was done to assess the relationships among the actual time outcomes, the perceived acceptability of time outcomes, and the levels of patient satisfaction. Correlation coefficients among the actual time outcomes, the perceived acceptability of time outcomes, and the patient satisfaction outcomes were obtained and reported.

## Results

### Sample Characteristics

Fifty-five patients agreed to participate, but 6 of them withdrew from the study without reporting reasons for withdrawal. Therefore, the data on 49 patients were used in the analysis. Table 1 presents the demographic characteristics of the participating patients.

**Table 1.** Patient Demographic Characteristics (n = 49).

Characteristics	
Age, y	
Mean (SD)	37.1 (13.1)
Median	32
Minimum	20
Maximum	73
Gender, n (%)	
Male/female	17 (35)/32 (65)
Self-reported health status, n (%)	
Poor	9 (18.4)
Fair	18 (36.7)
Good	21 (42.9)
Very good	1 (2)
Excellent	0 (0)
Highest education level attained, n (%)	
Primary school	1 (2)
High school	8 (16.4)
Associate degree	10 (20.4)
Bachelor’s degree	24 (49)
Master’s degree	5 (10.2)
Doctorate’s degree	1 (2)
Frequency of hospital visits, n (%)	
Several times a week	1 (2)
Once a week	2 (4.1)
Several times a month	9 (18.4)
Once a month	11 (22.4)
Several times a year	17 (34.7)
Once a year or less frequent	9 (18.4)

### Actual Waiting Time, Actual Service Time, and Actual Visit Duration

Table 2 presents descriptive statistics for the actual time outcomes. The results show that on average, 89.4% of the time involved in the hospital visit was spent waiting, and only 10.6% was spent receiving care services.

**Table 2.** Actual Time Spent on Waiting, Service, and Total Visitation (in Minutes).

Time outcomes	Mean	SD	Median	Minimum	Maximum
Actual waiting time	150.5	55.1	136.5	39.2	272.3
Actual service time	17.8	13.5	17.8	1.7	62.4
Actual visit duration	168.3	57.9	165.2	50.1	292.1

### Perceived Acceptability of Time Outcomes and Satisfaction With Care

Table 3 presents descriptive statistics for the patients’ perception and satisfaction outcomes, with higher scores representing more positive perceptions or higher levels of satisfaction. The patients’ responses on QPP-treatment waiting time (one of the indicators for perceived acceptability of waiting time) ranged from 1.0 to 5.0, with an average of 3.0. The participants’ responses regarding the other indicator for perceived acceptability of waiting time, VSQ-waiting time, ranged from 1.0 to 3.0, with an average of 1.7. The patients’ responses on both of the indicators for perception of service time ranged from 1.0 to 4.0, with an average of 2.5 for PSQ-time spent with doctor, and an average of 2.4 for VSQ-service time.

**Table 3.** Descriptive Statistics for Patient Perception and Satisfaction Outcomes.

Perception and satisfaction outcomes		Mean	SD	Median	Min	Max
QPP	QPP-physical caring	3.3	0.7	3.0	2.0	5.0
	QPP-medical care	3.4	0.7	3.5	2.0	5.0
	QPP-treatment waiting time	3.0	1.1	3.0	1.0	5.0
	*Dimension: Medical-technical competence*	3.2	0.6	3.2	2.3	4.3
	QPP-Physical-technical conditions	3.7	0.8	4.0	1.0	5.0
	*Dimension: Physical-technical conditions*	3.7	0.8	4.0	1.0	5.0
	QPP-information before procedures	3.7	0.7	4.0	2.0	5.0
	QPP-information after procedures	3.5	0.8	3.5	2.0	5.0
	QPP-responsible persons	2.8	0.8	3.0	1.0	5.0
	QPP-participation	3.2	0.9	3.0	2.0	5.0
	QPP-commitment (doctors)	3.8	0.8	4.0	1.0	5.0
	QPP-commitment (nurses and assistant nurses)	3.5	0.7	3.0	2.0	5.0
	QPP-empathic and personal (doctors)	3.6	0.7	4.0	2.0	5.0
	QPP-empathic and personal (nurses and assistant nurses)	3.2	0.9	3.0	2.0	5.0
	QPP-respect (doctors)	3.8	0.7	4.0	2.0	5.0
	QPP-respect (nurses and assistant nurses)	3.5	0.8	3.0	2.0	5.0
	*Dimension: Identity-oriented approach*	3.4	0.6	3.4	2.3	4.6
	QPP-secluded environment	3.1	0.9	3.5	1.0	5.0
	QPP-general atmosphere	3.4	0.8	4.0	2.0	5.0
	QPP-family and friends	3.4	0.6	3.0	2.0	5.0
	QPP-routines	3.3	0.8	3.0	2.0	5.0
	*Dimension: Sociocultural atmosphere*	3.3	0.6	3.2	2.0	4.4
PSQ	PSQ-general satisfaction	3.0	0.7	3.0	1.5	4.5
	PSQ-technical quality	3.5	0.6	3.5	2.0	4.8
	PSQ-interpersonal manner	3.4	0.6	3.5	2.0	4.5
	PSQ-communication	3.5	0.8	3.5	2.0	5.0
	PSQ-financial aspects	3.5	0.7	3.5	2.0	5.0
	PSQ-time spent with doctor	2.5	0.7	2.5	1.0	4.0
	PSQ-accessibility and convenience	2.5	0.7	2.3	1.5	4.0
VSQ	VSQ-appointment convenience	2.1	0.9	2.0	1.0	5.0
	VSQ-location convenience	2.8	0.8	3.0	1.0	5.0
	VSQ-phone accessibility	2.2	0.7	2.0	1.0	4.0
	VSQ-waiting time	1.7	0.7	2.0	1.0	3.0
	VSQ-service time	2.4	0.9	2.0	1.0	4.0
	VSQ-explanation	2.7	0.8	2.0	2.0	5.0
	VSQ-technical skills	3.0	0.8	3.0	2.0	5.0
	VSQ-personal manners	3.0	1.0	3.0	1.0	5.0
	VSQ-visit overall	2.7	0.8	3.0	2.0	5.0
	*VSQ (average of the 9 VSQ aspects)*	2.5	0.6	2.2	1.6	3.8

*Note.* QPP = Quality From the Patient’s Perspective; PSQ = Patient Satisfaction Questionnaire; VSQ = Visit-Specific Satisfaction Questionnaire.

### Correlation Analysis

Table 4 presents the correlations of the actual time outcomes and the perceptions of time outcomes with the levels of satisfaction with care.

**Table 4.** Correlation of Actual Time Outcomes and Perceived Acceptability of Time Outcomes With the Levels of Patient Satisfaction With Care.

Aspect/dimension of satisfaction with care	Actual time outcomes			Perceived acceptability of time outcomes			
	Actual waiting time	Actual service time	Actual visit duration
				Perceived acceptability of waiting time		Perceived acceptability of service time	
				QPP-treatment waiting time	VSQ-waiting time	PSQ-time spent with doctor	VSQ-service time
QPP-physical caring	−0.12	−0.09	−0.14	0.18	0.20	0.34*	0.43**
QPP-medical care	−0.02	−0.19	−0.07	0.19	0.24	0.27	0.43**
QPP-treatment waiting time	−0.43**	−0.04	−0.42**	−	0.51***	0.34*	0.30*
*Dimension: Medical-technical competence*	−0.24	−0.16	−0.27	0.63***	0.44**	0.42**	0.53***
*Dimension: Physical-technical conditions*	−0.03	−0.08	−0.05	0.34*	0.25	0.17	0.47***
QPP-information before procedures	0.04	−0.15	0.00	0.28	0.10	0.23	0.34*
QPP-information after procedures	−0.14	−0.33*	−0.21	0.34*	0.33*	0.42**	0.51***
QPP-responsible persons	−0.08	0.07	−0.06	0.29*	0.48***	0.53***	0.49***
QPP-participation	0.10	−0.27	0.03	0.30*	0.17	0.28*	0.43**
QPP-commitment (doctors)	0.05	−0.06	0.03	0.28	0.41**	0.50***	0.67***
QPP-commitment (nurses and assistant nurses)	0.13	−0.08	0.10	0.21	0.24	0.41**	0.55***
QPP-empathic and personal (doctors)	−0.10	−0.25	−0.15	0.37**	0.51***	0.43**	0.54***
QPP-empathic and personal (nurses and assistant nurses)	−0.06	−0.28	−0.12	0.32*	0.33*	0.34*	0.45**
QPP-respect (doctors)	0.06	−0.09	0.03	0.24	0.31*	0.64***	0.72***
QPP-respect (nurses and assistant nurses)	0.18	−0.11	0.15	0.30*	0.16	0.43**	0.46**
*Dimension: Identity-oriented approach*	0.00	−0.21	−0.05	0.41**	0.44**	0.58***	0.70***
QPP-secluded environment	−0.02	−0.18	−0.06	0.40**	0.31*	0.33*	0.36*
QPP-general atmosphere	−0.18	−0.08	−0.19	0.35*	0.41**	0.25	0.20
QPP-family and friends	−0.08	−0.05	−0.09	0.42**	0.37**	0.41**	0.40**
QPP-routines	−0.01	−0.13	−0.04	0.24	0.41**	0.25	0.40**
*Dimension: Sociocultural atmosphere*	−0.08	−0.17	−0.12	0.48***	0.47***	0.40**	0.45**
PSQ-general satisfaction	−0.07	−0.03	−0.07	0.45**	0.43**	0.64***	0.65***
PSQ-technical quality	0.02	−0.16	−0.02	0.31*	0.45**	0.64***	0.64***
PSQ-interpersonal manner	0.03	−0.01	0.02	0.38**	0.49***	0.70***	0.60***
PSQ-communication	0.30*	0.06	0.30*	0.19	0.38**	0.65***	0.64***
PSQ-financial aspects	−0.02	−0.23	−0.08	0.25	0.28*	0.28*	0.58***
PSQ-time spent with doctor	0.05	0.06	0.06	0.34*	0.48***	−	0.62***
PSQ-accessibility and convenience	−0.25	−0.19	−0.28	0.32*	0.44**	0.56***	0.52**
VSQ-appointment convenience	−0.18	−0.14	−0.21	0.39**	0.37**	0.18	0.36*
VSQ-location convenience	−0.29*	−0.12	−0.31*	0.30*	0.33*	0.05	0.40**
VSQ-phone accessibility	−0.33*	−0.09	−0.33*	0.34*	0.40**	0.18	0.36*
VSQ-waiting time	−0.30*	−0.20	−0.33*	0.51***	−	0.48***	0.47***
VSQ-service time	−0.03	−0.06	−0.04	0.30*	0.47***	0.62***	−
VSQ-explanation	−0.11	−0.12	−0.14	0.35*	0.43**	0.63***	0.80***
VSQ-technical skills	0.08	−0.06	0.06	0.29*	0.43**	0.52***	0.67***
VSQ-personal manners	0.14	0.03	0.14	0.25	0.38**	0.54***	0.74***
VSQ-visit overall	−0.09	−0.17	−0.12	0.40**	0.60***	0.50***	0.67***
*VSQ (average of the 9 VSQ aspects)*	−0.15	−0.13	−0.18	0.46***	0.65***	0.56***	0.83***

*Note.* QPP = Quality From the Patient’s Perspective; PSQ = Patient Satisfaction Questionnaire; VSQ = Visit-Specific Satisfaction Questionnaire.

* *P* < .05. **P < .01. ***P < .001.

#### Actual waiting time

Actual waiting time was negatively correlated with perceived acceptability of waiting time (*r* = –0.43 for QPP-treatment waiting time; *r* = –0.3 for VSQ-waiting time). In addition, actual waiting time was negatively correlated with VSQ-location convenience (*r* = –0.29) and VSQ-phone accessibility (*r* = –0.33), and it was positively correlated with PSQ-communication (*r* = –0.3).

#### Actual service time

Actual service time was significantly correlated only with QPP-information after procedures (*r* = –0.33).

#### Actual visit duration

Actual visit duration was negatively correlated with QPP-treatment waiting time (*r* = –0.42), VSQ-waiting time (*r* = –0.33), VSQ-location convenience (*r* = –0.31), and VSQ-phone accessibility (*r* = –0.33). Actual visit duration was also positively correlated with PSQ-communication (*r* = 0.30).

#### Perceived acceptability of waiting time

The 2 aspects that we used to indicate perceived acceptability of waiting time, namely, QPP-treatment waiting time and VSQ-waiting time, were significantly correlated (*r* = 0.51).

QPP-treatment waiting time was positively correlated with all 4 dimensions of QPP: medical-technical competence (*r* = 0.63), physical-technical conditions (*r* = 0.34), identity-oriented approach (*r* = 0.41), and sociocultural atmosphere (*r* = 0.48). Also, QPP-treatment waiting time was significantly correlated with PSQ-general satisfaction (*r* = 0.45), PSQ-technical quality (*r* = 0.31), PSQ-interpersonal manner (*r* = 0.38), PSQ-time spent with doctor (*r* = 0.34), and PSQ-accessibility and convenience (*r* = 0.32). We also found that QPP-treatment waiting time was positively correlated with all VSQ aspects except for VSQ-personal manners (*r* ranges from 0.29 to 0.51).

VSQ-waiting time was positively correlated with QPP-medical-technical competence (*r* = 0.44), QPP-identity-oriented approach (*r* = 0.44), and QPP-sociocultural atmosphere (*r* = 0.47). Also, VSQ-waiting time was significantly correlated with all aspects of PSQ (*r* ranges from 0.28 to 0.49) and all of the VSQ aspects (*r* ranges from 0.33 to 0.83).

#### Perceived acceptability of service time

The 2 indicators of perceived acceptability of service time (ie, PSQ-time spent with a doctor and VSQ-service time) were both positively correlated with the following aspects of satisfaction regarding care (as determined in the PSQ assessment): general satisfaction (*r* = 0.64 and 0.65), technical quality (*r* = 0.64 and 0.64), interpersonal manner (*r* = 0.70 and 0.60), communication quality (*r* = 0.65 and 0.64), financial aspects (*r* = 0.28 and 0.58), and accessibility and convenience (*r* = 0.56 and 0.52). For the QPP measures, at least one of the indicators of perceived acceptability of service time was positively correlated with the following QPP dimensions: medical-technical competence (*r* = 0.42 and 0.53), physical-technical conditions (*r* = 0.47), identity-oriented approach (*r* = 0.58 and 0.70) and sociocultural atmosphere (*r* = 0.40 and 0.45). In addition, perceived acceptability of service time was positively correlated with perceived acceptability of waiting time (*r* = 0.3 and 0.34 for QPP-treatment waiting time, and *r* = 0.47 and 0.48 for VSQ-waiting time).

## Discussion

This study examined how long the patients waited, how much time they spent receiving care services, and how much time they spent in total during their visits to an endocrinology outpatient department of a major hospital in China. The patients’ perceived acceptability of how long they waited and how much time they spent receiving health care services was also considered. We assessed the relationships between the time outcomes and the patients’ satisfaction with care received. We identified several factors that might affect the patients’ perceived acceptability of waiting time and service time, so that we could provide feasible suggestions to deal with the patients’ perceptions regarding these 2 types of time outcomes, and thus mitigate the patient dissatisfaction resulting from long waiting periods.

### Actual Waiting Time

Our findings regarding the relationship between how long patients waited and their perceived acceptability of the wait length showed that patients who waited longer perceived their length of time waiting as less acceptable. Moreover, the inverse relationships of actual waiting with VSQ-location convenience and VSQ-phone accessibility suggested that long waits could negatively affect the patients’ perceptions regarding the convenience and accessibility of the hospital. Another finding regarding the positive relationship between actual waiting and PSQ-communication indicated that, contrary to our expectation, patients who experienced longer waits appeared to feel less ignored by their doctors during their periods of communication with medical professionals. Also, these patients reported feeling more satisfied with the explanations they received regarding medical tests. One possible explanation for this response could be that while the patients were waiting for their care services, they were able to consult nurses in the waiting area for information about their medical tests or the treatments that they were going to receive. Patients who waited longer might have had more opportunities to receive such information during their waits, which may have improved their satisfaction with the attention they received.

### Perceived Acceptability of Waiting Time

We found that the 2 QPP dimensions (ie, identity-oriented approach and sociocultural atmosphere) were related to the patients’ perceptions of waiting. These 2 dimensions concerned various aspects of satisfaction with care, including the quality and amount of information provided regarding the services that they were about to receive and the health professionals who would be responsible for those services. The dimensions were also related to the degree to which patients were allowed to participate in making decisions that applied to their care, the degree of understanding that doctors and nurses had for the patients’ difficulties with the illness conditions, the level of respect that the staff showed toward the patients, the privacy of the spaces provided for patients to talk to the health professionals, the pleasantness of the atmosphere in the wards/clinics, and the ways that family members or friends who accompanied the patients were treated. It appeared that enhancements in these aspects of the experience could help to mitigate the patients’ dissatisfaction with waiting times. This suggestion was also supported by the positive correlations between the patients’ perceived acceptability of waiting times and the aspects of PSQ-interpersonal manner, PSQ-communication, VSQ-explanation, and VSQ-personal manner.

Hence, our suggestions for improving the patients’ perceptions of waiting times include offering the patients useful information on how their tests and treatments will be done, giving adequate explanations of the test results, informing the patients clearly as to which health care professionals are responsible for their care, encouraging doctors and nurses to show empathy and respect toward patients, providing patients with private space when needed, creating a pleasant atmosphere in the hospital, and encouraging the staff to treat the patients’ accompanying family members or friends well.

### Actual Service Time and Perceived Acceptability of Service Time

Actual service time was not significantly correlated with perceived acceptability of service time. This lack of correlation may have occurred for 2 reasons. First, the patients might not have had a strong sense of time as they were being treated. Their attention might have been focused on their treatments or on their interactions with the doctors and nurses. In contrast, the patients might have had a stronger sense of time while they were waiting, especially if they were not occupied or if their attention was not diverted from waiting. These factors may also explain why the correlation between the perceived acceptability of waiting time and the actual waiting time was much greater than the correlation between the perceived acceptability of service time and the actual service time. Second, we notice that the actual service time of our patients ranged from 1.7 to 62.4 minutes, with an average of 17.8 minutes. Therefore, the service time might have been generally too short for patients to experience much difference between the actual time they spent receiving care services and their perception of that time period. In contrast, the actual waiting time of our patients ranged from 39.2 to 272.3 minutes, with a mean of 150.5 minutes. These waiting times were long enough that the patients tended to form strong perceptions of how long they had waited. The negative correlation between the actual service time and the PSQ-information after procedures was also contrary to our intuition. This finding suggested that patients who spent more time with their health professionals tended to be less satisfied with the information provided about self-care, or with the results of the tests or treatments they received.

At least one of the indicators regarding perceived acceptability of service time was positively correlated with medical-technical competence, physical-technical conditions, identity-oriented approach, and sociocultural atmosphere, as indicated in the QPP measure. This set of findings might suggest that the contents and effectiveness of the health care provided have a greater influence on the patients’ perceived acceptability of service time than the actual amount of time that the health care professionals spent with them.

### Limitations and Future Work

This study has several limitations. We evaluated patients’ perceived acceptability of time outcomes through their responses to questionnaire items that investigated their levels of satisfaction with those outcomes. However, the actual data regarding the amounts of time that the participants thought they spent in waiting or receiving health care services were not collected. We were unable to assess the relationships between the lengths of perceived time and the lengths of actual time spent waiting or receiving care. In future work, it would be meaningful to ask the participants to report their perceived time outcomes in a quantitative way, so that we can assess the deviations between patients’ perceived time outcomes and the actual time outcomes. Such an approach could also allow us to investigate the directions of any deviations, discuss the factors that affect such deviations, and make suggestions to better manage the patients’ perceived time outcomes. Also, the limited sample size of the study does not allow us to conduct subgroup analyses to further understand the factors related to patient dissatisfaction (eg, why patients who spent more time with health care professionals tended to be less satisfied toward the information provided about the care services they received). Moreover, participant bias that could emerge due to the situation that the participants were followed and observed by research assistants might affect the results. As the patients knew that they were being studied, some patients might exaggerate their dissatisfaction with the waiting time, especially for those who had negative perceptions of waiting for care services before. However, the issue may not be easily addressed in direct field observations and questionnaire study.

## Conclusions

Patients who experienced longer waits tended to consider their health care services as less accessible and their waiting times as less acceptable. Also, spending a longer time in receiving care services did not always correlate with a more positive perception of the services. Our study shows that patients who actually spent longer periods of time receiving care services did not perceive that they had spent more time in those activities, and they were no more satisfied with the service they received than those who spent less time receiving such services. Accordingly, we suggest that the effectiveness of the health care service and the attitudes of the caregivers actually matter more than the length of the treatment. Although the problem of long waiting periods is difficult to solve through actually reducing the waiting times, it may be possible to better manage how patients feel about the amount of time they have to wait and the amount of time they spend receiving care services. The patients’ feelings about waiting may be mitigated through several patient-centered strategies, such as providing patients with useful information about the care services they are going to receive and the health professionals who will provide those services. Doctors and nurses can also be encouraged to offer respect and empathy to patients, to provide the patients with private spaces to talk to doctors when needed, and to treat the patients’ accompanying family members or friends in a friendly way.

We conclude that although actually reducing wait times may not always be possible (due to lack of resources or staffing limitations), it remains possible to improve some patient-centered aspects of care services, which can mitigate the patients’ dissatisfaction with waiting periods and enable more positive perceptions regarding the services they receive.

## Appendix

**Table A1.** QPP Dimensions, Aspects, and Items.

Dimension	Aspect	Item #	Item (rated on a 5-point scale ranging from 1 [strongly disagree] to 5 [strongly agree])
Medical-technical competence	QPP-physical caring	QPP 1	I receive the best possible physical care.
	QPP-medical caring	QPP 2	I receive the best possible medical care.
		QPP 3	I receive effective pain relief.
	QPP-treatment waiting time	QPP 4	I receive examinations and treatments within acceptable waiting times.
Physical-technical conditions	QPP-physical-technical conditions	QPP 5	I had access to the apparatus and equipment that was necessary for my medical care.
Identity-oriented approach	QPP-information before procedures	QPP 6	I receive useful information on how examinations and treatments would take place.
	QPP-information after procedures	QPP 7	I receive useful information on the results on examination and treatments.
		QPP 8	I receive useful information on self-care (eg, how I should take care of myself).
	QPP-responsible persons	QPP 9	I receive useful information on which doctors were responsible for my medical care.
		QPP 10	I receive useful information on which nurses were responsible for my nursing care.
	QPP-participation	QPP 11	I had good opportunity to participate in the decisions that applied to my care.
	QPP-commitment (doctors)	QPP 12	The doctors showed commitment (care about me).
	QPP-commitment (nurses and assistant nurses)	QPP 13	The nurses and assistant nurses showed commitment (cared about me).
	QPP-empathic and personal (doctors)	QPP 14	The doctors seemed to understand how I experienced my situation.
	QPP-empathic and personal (nurses and assistant nurses)	QPP 15	The nurses and assistant nurses seemed to understand how I experienced my situation.
	QPP-respect (doctors)	QPP 16	The doctors were respectful toward me.
	QPP-respect (nurses and assistant nurses)	QPP 17	The nurses and assistant nurses were respectful toward me.
Sociocultural atmosphere	QPP-secluded environment	QPP 18	I talked to the doctors in private when I wanted to.
		QPP 19	I talked to the nurses in private when I wanted to.
	QPP-general atmosphere	QPP 20	There was a pleasant atmosphere on the ward.
	QPP-family and friends	QPP 21	My relatives and friends were treated well.
	QPP-routines	QPP 22	My care was determined at my own request and needs rather than staff procedures.

*Note.* QPP = Quality From the Patient’s Perspective.

**Table A2.** PSQ Aspects and Items.

Aspect	Item #	Item (rated on a 5-point scale ranging from 1 [strongly disagree] to 5 [strongly agree])
PSQ-general satisfaction	PSQ 3	The medical care I have been receiving is just about perfect.
	PSQ 17	I am dissatisfied with some things about the medical care I received.
PSQ-technical quality	PSQ 2	I think my doctor’s office has everything needed to provide complete medical care.
	PSQ 4	Sometimes doctors make me wonder if their diagnosis is correct.
	PSQ 6	When I go for medical care, they are careful to check everything when treating and examining me.
	PSQ 14	I have some doubts about the ability of the doctors who treat me.
PSQ-interpersonal manner	PSQ 10	Doctors act too businesslike and impersonal toward me.
	PSQ 11	My doctors treat me in a very friendly and courteous manner.
PSQ-communication	PSQ 1	Doctors are good about explaining the reason for medical tests.
	PSQ 13	Doctors sometimes ignore what I tell them.
PSQ-financial aspects	PSQ 5	I feel confident that I can get the medical care I need without being set back financially.
	PSQ 7	I have to pay for more of my medical care than I can afford.
PSQ-time spent with doctor	PSQ 12	Those who provide my medical care sometimes hurry too much when they treat me.
	PSQ 15	Doctors usually spend plenty of time with me.
PSQ-accessibility and convenience	PSQ 8	I have easy access to the medical specialists I need.
	PSQ 9	Where I get medical care, people have to wait too long for emergency treatment.
	PSQ 16	I find it hard to get an appointment for medical care right away.
	PSQ 18	I am able to get medical care whenever I need it.

*Note.* The scores of items 4, 7, 9, 10, 12-14, 16, and 17 were reversed during the analysis, so that higher scores for these items represented higher patient satisfaction with those aspects of care. PSQ = Patient Satisfaction Questionnaire.

**Table A3.** VSQ Aspects and Items.

Aspect	Item #	Item (rated on a 5-point scale ranging from 1 [poor] to 5 [excellent])
VSQ-appointment convenience	VSQ 1	Your waiting time to get an appointment.
VSQ-location convenience	VSQ 2	Convenience of the location of the hospital.
VSQ-phone accessibility	VSQ 3	Getting through to the hospital by phone.
VSQ-waiting time	VSQ 4	Length of time waiting at the hospital.
VSQ-service time	VSQ 5	Time spent with the physician/health care professional you saw.
VSQ-explanation	VSQ 6	Explanation of what was done for you.
VSQ-technical skills	VSQ 7	Technical skills (thoroughness, carefulness, competence) of the physician/health care professional you saw.
VSQ-personal manners	VSQ 8	The personal manner (courtesy, respect, sensitivity, friendliness) of the person you saw.
VSQ-visit overall	VSQ 9	The visit overall.
Average of the 9 VSQ aspects	Item #	Item
VSQ	VSQ 1-9	Average of the 9 VSQ aspects

*Note.* VSQ = Visit-Specific Satisfaction Questionnaire.
